# Distinguishing Early from Late Mild Cognitive Impairment Using Magnetic Resonance Free-Water Diffusion Tensor Imaging

**DOI:** 10.3390/neurosci6010008

**Published:** 2025-01-18

**Authors:** Maurizio Bergamino, Molly M. McElvogue, Ashley M. Stokes, Alzheimer’s Disease Neuroimaging Initiative

**Affiliations:** Barrow Neuroimaging Innovation Center, Barrow Neurological Institute, Phoenix, AZ 85013, USA; maurizio.bergamino@barrowneuro.org (M.B.); molly.mcelvogue@commonspirit.org (M.M.M.)

**Keywords:** early mild cognitive impairment, late mild cognitive impairment, diffusion tensor imaging, free-water DTI, dementia

## Abstract

Mild Cognitive Impairment (MCI) is a transitional stage between normal aging and Alzheimer’s disease. Differentiating early MCI (EMCI) from late MCI (LMCI) is crucial for early diagnosis and intervention. This study used free-water diffusion tensor imaging (fw-DTI) to investigate white matter differences and voxel-based correlations with Mini–Mental State Examination (MMSE) scores. Data from the Alzheimer’s Disease Neuroimaging Initiative included 476 healthy controls (CN), 137 EMCI participants, and 62 LMCI participants. Significant MMSE differences were found between the CN and MCI groups, but not between EMCI and LMCI. However, distinct white matter changes were observed: LMCI showed a higher f-index and lower fw-fractional anisotropy (fw-FA) compared to EMCI in several white matter regions. These findings indicate specific white matter tracts involved in MCI progression. Voxel-based correlations between fw-DTI metrics and MMSE scores further supported these results. In conclusion, this study provides crucial insights into white matter changes associated with EMCI and LMCI, offering significant implications for future research and clinical practice.

## 1. Introduction

Mild Cognitive Impairment (MCI) is a transitional stage between normal age-related cognitive decline and the more severe decline associated with dementia. MCI is characterized by subtle changes in memory, thinking, visuospatial abilities, and other cognitive functions [1,2]. These noticeable changes exceed what is expected for a person’s age and education level. Studies have shown that individuals with MCI progress to probable Alzheimer’s disease (AD) at a significantly higher rate compared to older adults without memory problems [3,4]. Additionally, episodic memory performance in MCI declines at a faster rate than that of healthy aging individuals but less rapidly than in those diagnosed with mild AD [5]. Currently, there is no cure for MCI; therefore, studying its characteristics, risk factors, and progression might be helpful for the early detection of and intervention in cognitive decline in an aging population.

The Alzheimer’s Disease Neuroimaging Initiative (ADNI; https://adni.loni.usc.edu/) was created in 2004 as a partnership to develop clinical, imaging, genetic, and biochemical biomarkers for AD and dementia. This database has been instrumental in advancing our understanding of AD, leading to the development of new treatments for people with dementia across multiple sites [6]. The ADNI database recognizes two subcategories of MCI: “early MCI” (EMCI) and “late MCI” (LMCI). While both stages meet the conventional criteria for MCI, EMCI is believed to represent an earlier point in the clinical spectrum of the disease [7]. Distinguishing between EMCI and LMCI is critical for understanding disease trajectory, tailoring interventions, and stratifying patients for clinical trials, as well as supporting patient and caregiver care plans.

As part of ADNI data collection, magnetic resonance imaging (MRI) data are collected in a subset of individuals. For instance, diffusion MRI (dMRI) is a non-invasive imaging technique that utilizes the movement of water molecules to study the microstructural organization of white matter tracts [8,9]. Through its related metrics, dMRI characterizes the diffusion of water molecules within and around structures like white matter fibers and cell bodies [10]. Diffusion tensor imaging (DTI) is a specific dMRI analysis method that measures the anisotropy of diffusion, revealing information about the microstructural organization of white matter tracts [11,12]. However, despite its power, DTI has several limitations that can lead to potential misinterpretations of the resulting metrics. For example, DTI assumes that water diffusion within a brain voxel follows a Gaussian distribution [13]. This assumption may not hold true in areas with complex tissue microstructures, such as regions containing multiple tissue types; for this reason, DTI cannot characterize complex fiber structures or crossing fibers [14].

DTI is also susceptible to partial volume effects (PVEs), where the measured diffusion within a voxel reflects contributions from both tissue and free water (fw), leading the DTI metrics to reflect a weighted average of multiple diffusion components within a single voxel [15]. PVEs can significantly impact DTI measurements, particularly in dementia and AD. For instance, AD is characterized by neuronal loss, which leads to increased cerebrospinal fluid (CSF) and fw across the brain. This increase in extracellular fw can distort DTI-derived metrics, making it difficult to accurately quantify the microstructural integrity of white matter tracts [16]. To overcome the effects of extracellular fw on DTI measurements, an fw correction algorithm has been developed to quantify and remove the contribution of extracellular fw from the DTI signal, allowing for a more accurate assessment of white matter microstructure [17].

Several studies have investigated the use of fw-DTI in individuals with MCI. While single-shell fw-DTI can provide valuable insights, particularly in differentiating between various neurodegenerative conditions, it is important to acknowledge the inherent limitations of using single-shell diffusion MRI data. These limitations include potential instability and reduced specificity, especially in the presence of pathology, as highlighted by recent research [18]. Despite this limitation, Dumont et al. developed a simple yet powerful whole-brain fw measure designed for clinical application and potential use as an outcome measure in clinical trials [19]. This approach allowed them to identify white matter differences between AD, MCI, and cognitively normal (CN) individuals. In a separate investigation, Schumacher et al. utilized an fw-DTI model to explore cholinergic projection systems in individuals with dementia with Lewy bodies (DLB), AD, and MCI [20]. Their findings underscored the unique patterns of cholinergic pathway degeneration in AD and DLB, emphasizing the potential significance of this pathway in the manifestation of DLB-associated visual hallucinations.

In this study, we employed fw-DTI to analyze differences in the fw index (f-index) and white matter microstructure (through the fw-fractional anisotropy (fw-FA)) between groups of individuals with EMCI and LMCI from the ADNI dataset. Furthermore, we compared each of these groups with a cohort of cognitively normal (CN) individuals. To elucidate potential associations between neuroimaging metrics and cognitive function, we explored voxel-based correlations between fw-DTI metrics and the Mini–Mental State Examination (MMSE) [21]. Our primary focus was to understand whether fw-DTI metrics can distinguish individuals with LMCI and EMCI from CN. Additionally, we explored whether these metrics could differentiate further between EMCI and LMCI within the spectrum of MCI.

## 2. Materials and Methods

### 2.1. Participants and Cognitive Measures

This study included 476 CN subjects (284 females, mean age 72.8 ± 8.0 years), 137 subjects with EMCI (55 females, mean age 74.0 ± 7.6 years), and 62 subjects with LMCI (28 females, mean age 73.4 ± 6.0 years). The EMCI and LMCI groups were categorized based on the ADNI criteria. Inclusion criteria were participants between the ages of 55 and 90 years with available 3T dMRI data.

From ADNI, we obtained cognitive test scores for all participants. The MMSE is a widely used cognitive screening tool to assess mental status, particularly for detecting dementia. Total scores on the MMSE range from 0 to 30, with higher scores indicating better cognitive function. A score of 24 or below is generally considered indicative of cognitive impairment [21]. In this study, we investigated the voxel-based correlations of fw-DTI metrics with MMSE scores in the combined EMCI + LMCI group and in both the EMCI and LMCI groups separately.

Additionally, we also included the ratings of the Geriatric Depression Scale (GDS), a screening tool for assessing depression in older adults [22]; the global Clinical Dementia Rating (CDR), a widely used tool for assessing dementia severity and progression [23]; and the Functional Activities Questionnaire (FAQ), a standardized tool used to evaluate a person’s ability to perform daily living activities [24]. Table 1 shows the complete demographics, scanner information, and clinical characteristics of the study participants.

### 2.2. MRI Acquisition

Data were downloaded between August and September 2024.

All participants underwent whole-brain MRI scanning at 3 Tesla. The scanner manufacturers used in this study were SIEMENS and General Electrics (GE); see Table 1 and Table 2. The dMRI data were downloaded from the ADNI database, and only single-shell dMRI data were analyzed. T1-weighted structural images were also obtained and were used for volumetric analysis.

For more specific information, refer to the ADNI webpage.

### 2.3. Data Pre-Processing 

The DICOM data obtained from the ADNI database were converted to the NIFTI format using the dcm2niix tool (v1.0.20240202, Columbus, OH 43210, USA, https://github.com/rordenlab/dcm2niix) and subsequently preprocessed using Mrtrix3 (v3.0.4, London, UK, https://www.mrtrix.org/) [25], FMRIB Software Library (v6.0.6.5, Oxford, UK, FSL) [26], and the Advanced Normalization Tools (ANTs) (v1.9, Philadelphia, PA 19104, USA, https://github.com/ANTsX/ANTs). The preprocessing of the dMRI data included denoising with dwidenoise [27] (MRtrix3), alignment, and eddy-current corrections using eddy [28] (FSL). The quality of the dMRI dataset was assessed using eddy QC tools. Instances of signal loss resulting from a subject’s movement coinciding with diffusion encoding were identified, and the affected slices were replaced by predictions generated through a Gaussian process. For this study, the quality control criteria were set as an average absolute volume-to-volume head motion of <3 mm or total outliers <5%.

Brain extraction from the b0 images was performed using dwi2mask (MRtrix3). Subsequently, all preprocessed dMRI images were resampled to a voxel size of 1.25 mm using mrgrid (MRtrix3). The computation of fw-corrected metrics, including fw-FA and the f-index, was conducted using a custom MATLAB script (v.R2023a, Natick, MA, USA, www.mathworks.com). All scripts used in this study are available upon reasonable request.

The harmonization of all fw-DTI metrics, to account for variations from different MRI scanners and dMRI acquisition protocols (batch id vector), was performed using ComBat (MATLAB v.R2023a, Baltimore, MD, USA, https://github.com/Jfortin1/ComBatHarmonization) [29] within a custom Matlab script. This process incorporated biological variables such as sex, age, and subject motion. This harmonization procedure is essential for large-scale studies involving data collected from multiple locations.

Following the harmonization process, all fw-DTI metrics underwent coregistration to MNI 2 mm space through an ANTs symmetric image normalization (SyN) coregistration algorithm [30]. All voxel-based statistical analyses were performed in MNI standard space.

Clusters identified as statistically different across groups were labeled according to the JHU DTI-based white-matter atlases [31,32].

Structural images were analyzed volumetrically using the ANTs toolkit. Brain extractions were performed with the antsBrainExtraction.sh script. Subsequently, brain segmentation into white matter, gray matter, and CSF was conducted using Atropos, an ANTs segmentation tool.

### 2.4. Statistical Analyses

Demographic and clinical characteristics, including age, MMSE, GDS, Global CDR, FAQ, motion, and outliers, are presented as the mean and standard deviation (SD) for each group. Group differences for age, for all cognitive tests (MMSE, GDS, CDR, and FAQ), and for regional brain volumes were evaluated using the Kruskal–Wallis test followed by the Dunn test corrected by the Bonferroni procedure due to non-normality (Shapiro–Wilk: *p* < 0.001 for all tests). Group differences in motion and outliers were assessed using the Kruskal–Wallis test due to non-normality (Shapiro–Wilk: *p* < 0.001 for all motion/outlier measurements).

For all fw diffusion metrics, voxel-based differences across groups were evaluated on the harmonized fw-DTI metrics using one-way analysis of covariance (ANCOVA) with a linear model function. Analyses were performed using an in-house script written in R (version 3.6.3, Vienna, Austria, www.r-project.org) and RStudio (version 1.3.1093, Boston, MA, USA, https://www.rstudio.com/categories/rstudio-ide/). Post hoc comparisons between groups were conducted using Tukey’s test.

To enhance clustering robustly without arbitrary thresholding and to account for multiple comparisons, the Threshold-Free Cluster Enhancement (TFCE) method [33] was used, incorporating a Family-Wise Error (FWE) rate correction with the Benjamini–Yekutieli (BY) procedure [34] at the 0.05 level (FWE < 0.05). This approach identified significant clusters of voxels with differential fw-DTI metrics across groups. Effect sizes were calculated for all analyses. Specifically, partial eta squared (η^2^) was used to estimate the effect size for ANCOVA, with η^2^ ≥ 0.073 indicating a large effect (⍺ = 0.05; power = 85%). For post hoc comparisons between groups, the Hedges’ g was employed (⍺ = 0.05; power = 85%) with a large effect classified as follows: CN vs. EMCI = |g| ≥ 0.29; CN vs. LMCI = |g| ≥ 0.41; EMCI vs. LMCI = |g| ≥ 0.47.

Receiver Operating Characteristic (ROC) analysis for the MMSE, f-index, and fw-FA within the significant clusters of group differences was conducted using a custom R script.

Within the EMCI and LMCI groups, a voxel-based linear model was performed in R to assess the correlations between the fw-FA/f-index and MMSE score. Due to the EMCI and LMCI sample size differences, Spearman’s rank correlation coefficient is reported (with moderate: >0.30; strong: >0.50; and very strong: >0.70 effect sizes) for each correlation.

## 3. Results

Two male CN participants, two female CN participants, one female EMCI participant, and two female LMCI participants were removed from the final analysis due to excessive motion.

Among the final included participants, no statistical differences were found for age (χ^2^ = 5.03; *p* = 0.081) across all groups. However, statistical differences were found for all cognitive tests (MMSE: χ^2^ = 74.57; *p* < 0.001; GDS: χ^2^ = 66.58; *p* < 0.001; Global CDR: χ^2^ = 198.72; *p* < 0.001; FAQ: χ^2^ = 651.58; *p* < 0.001).

Post hoc analysis identified specific cognitive tests that differentiated the groups: all cognitive domains distinguished CN from EMCI and LMCI and only FAQ distinguished EMCI from LMCI.

No statistical differences were found across the three groups for absolute motion (χ^2^ = 1.16; *p* = 0.561), relative motion (χ^2^ = 0.136; *p* = 0.934), and total outliers (χ^2^ = 3.226; *p* = 0.200). The statistical results for all cognitive scores, motion, outliers, and APOE status are reported in Table 1.

### 3.1. f-Index Results

**Figure 1:**
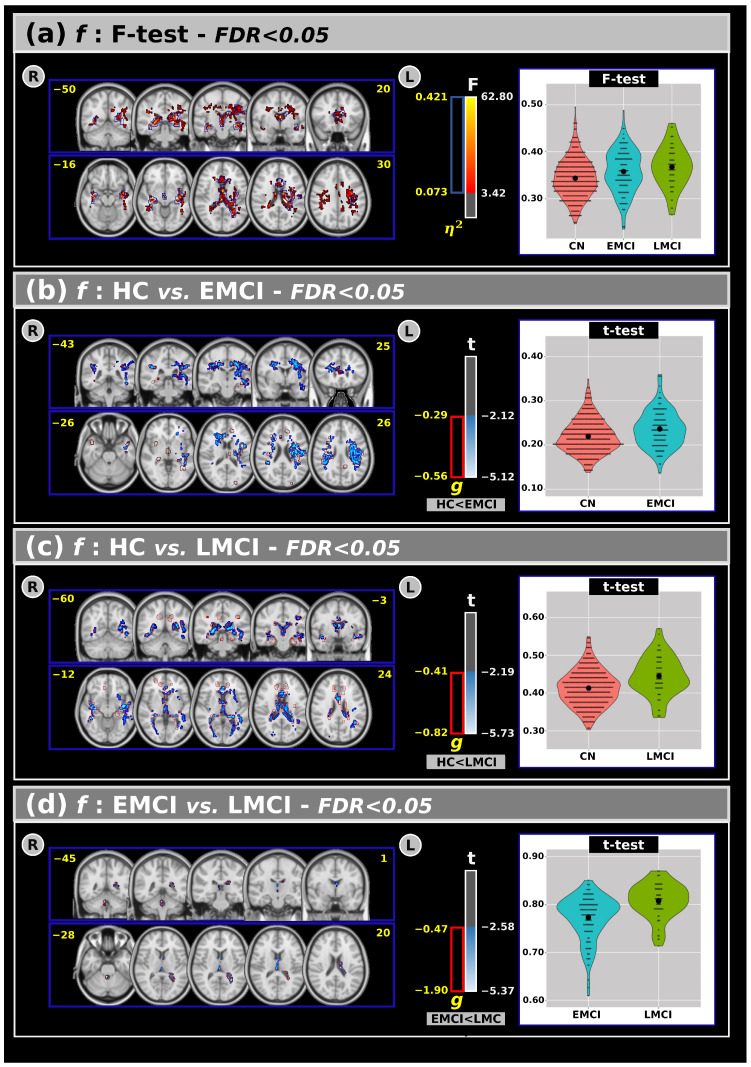
(**a**) The analysis of covariance revealed significant differences across groups for the f index, with the F-test and effect size outcomes at a False Discovery Rate (FDR) < 0.05. Subsequent post hoc comparisons using Tukey’s Honest Significant Difference (HSD) test showed significant differences in the t-values and effect sizes for the f-index between the (**b**) CN and EMCI, (**c**) CN and LMCI, and (**d**) EMCI and LMCI groups. Violin plots are presented to visualize the distribution of the mean f-values within the significant clusters for each group. R: right. L: left. CN: cognitively normal. EMCI: Early Mild Cognitive Impairment. LMCI: Late Mild Cognitive Impairment. g: effect size for the post hoc comparisons. η^2^: Effect size for the analysis of covariance (ANCOVA) model. vs: versus. The numbers in yellow are the MNI slices.

Figure 1a shows the ANCOVA results for the f-index across all groups, highlighting both F-test and effect size outcomes. The analysis revealed significant differences among the groups, indicating that individuals with EMCI and LMCI generally exhibited higher free-water values in several white matter areas compared to the CN group.

Panels (b), (c), and (d) present post hoc comparisons for the f-index. Panel (b) shows that the EMCI group had higher f-index values than the CN group in large clusters, including the corticospinal tracts, superior longitudinal fasciculus, genu of the corpus callosum, corona radiata, and superior fronto-occipital fasciculus. These findings were also supported by a large effect size.

In panel (c), both the t-test and effect size analyses revealed significant differences between the CN and LMCI groups in various white matter regions, such as the anterior thalamic radiation, forceps major/minor, corpus callosum, fornix, posterior thalamic radiation, and sagittal stratum, with the LMCI group showing higher f-index values compared to the CN group. Interestingly, panel (d) identified significant differences between the EMCI and LMCI groups (in both t-tests and effect size), primarily within the anterior thalamic radiation, forceps major, fornix, and left tapetum, where the LMCI group exhibited higher free-water values than the EMCI group.

An overview of the f-index results, including volumes and t-test outcomes at FDR < 0.05, is provided in Table 3.

### 3.2. FW-FA Results

**Figure 2:**
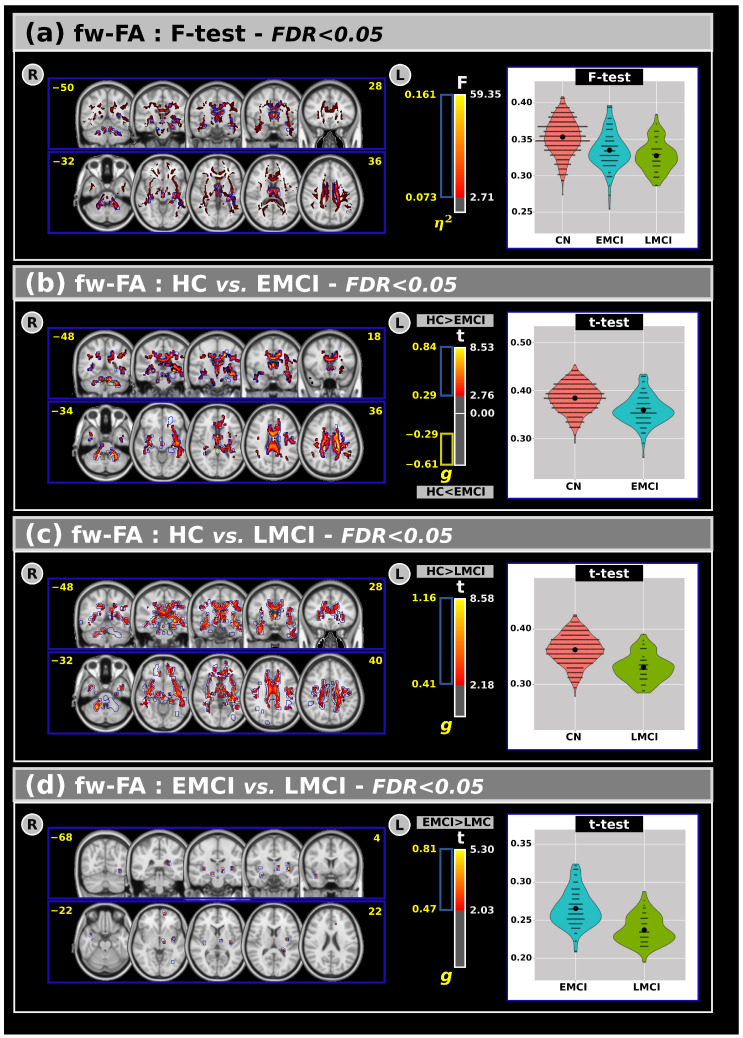
(**a**) The analysis of covariance revealed significant differences across groups for the free-water fractional anisotropy with F-test and effect size outcomes at FDR < 0.05. Subsequent post hoc comparisons using Tukey’s HSD test showed significant difference in t values and effect sizes for the free-water fractional anisotropy between (**b**) CN and EMCI, (**c**) CN and LMCI, and (**d**) EMCI and LMCI groups. Violin plots are presented to visualize the distribution of the mean free-water fractional anisotropy within the significant clusters for each group. R: Right. L: Left. CN: Cognitively Normal. EMCI: Early Mild Cognitive Impairment. LMCI: Late Mild Cognitive Impairment. g: Effect size for the post hoc comparisons. η^2^: Effect size for the Analysis of Covariance (ANCOVA) model. vs: Versus. The number in yellow are the MNI slices.

Figure 2a shows the ANCOVA results for the fw-FA metric across all groups. Statistically significant differences emerged among the groups, revealing that the CN group generally exhibited higher fw-FA values than the EMCI and LMCI groups in large clusters within the white matter.

Panels (b), (c), and (d) display post hoc comparisons for the fw-FA metric. In panel (b), both t-tests and effect size measures indicate significant differences between the CN and EMCI groups. Panel (c) shows that significant differences were also observed between the CN and LMCI groups in several white matter regions. Compared to CNs, individuals with EMCI and LMCI exhibited lower fw-FA values. Additionally, (panel (d)) a small but significant cluster of differences was found between the EMCI and LMCI groups, with lower fw-FA values in the LMCI group. These clusters were primarily located within the anterior thalamic radiation, right corticospinal tract, forceps minor, right uncinate fasciculus, external capsule, and the right fornix (cres)/stria terminalis.

An overview of the fw-FA results, including volumes and t-test outcomes at FDR < 0.05, is provided in Table 4.

### 3.3. ROC Analysis

**Figure 3:**
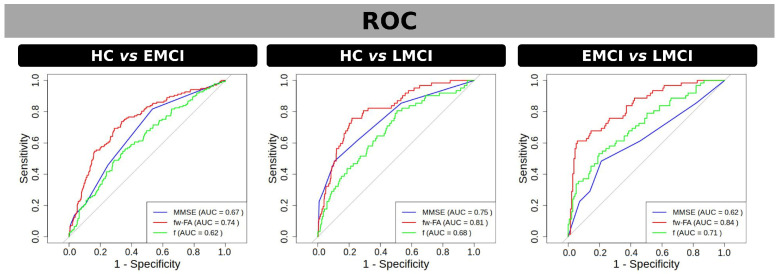
ROC curves for discriminating between groups using fw-FA (red) and f (green) within significant clusters of differences found by t-test, as well as using MMSE scores (blue). Panels represent group comparisons: CN vs. EMCI (**left**), CN vs. LMCI (**center**), and EMCI vs. LMCI (**right**). The area under the curve (AUC) values are displayed for each metric in the legend, indicating the discriminative performance of each measure. fw-FA consistently shows the highest AUC across all comparisons, highlighting its superior ability to differentiate between groups. Grey line in the ROC curves represents the line of no-discrimination.

The ROC analysis (Figure 3) was used to evaluate the ability of MMSE, fw-FA, and the f-index to discriminate the groups within significant clusters of differences found by fw-FA and the f-index. In the comparison between CN and EMCI, fw-FA demonstrated the highest discriminative power with an AUC of 0.74, followed by MMSE with an AUC of 0.67, while the f-index had a lower performance with an AUC of 0.62. For the CN vs. LMCI comparison, fw-FA showed the strongest discriminative ability with an AUC of 0.81, MMSE performed moderately with an AUC of 0.75, and the f-index had an AUC of 0.68. Finally, in the EMCI vs. LMCI comparison, fw-FA exhibited the highest discriminative power with an AUC of 0.84, the f-index had an AUC of 0.71, and MMSE showed the lowest performance with an AUC of 0.62. These results highlight the superior performance of fw-FA in distinguishing between groups across all comparisons.

### 3.4. Correlations Between dMRI Metrics and MMSE in Individuals with MCI

**Figure 4:**
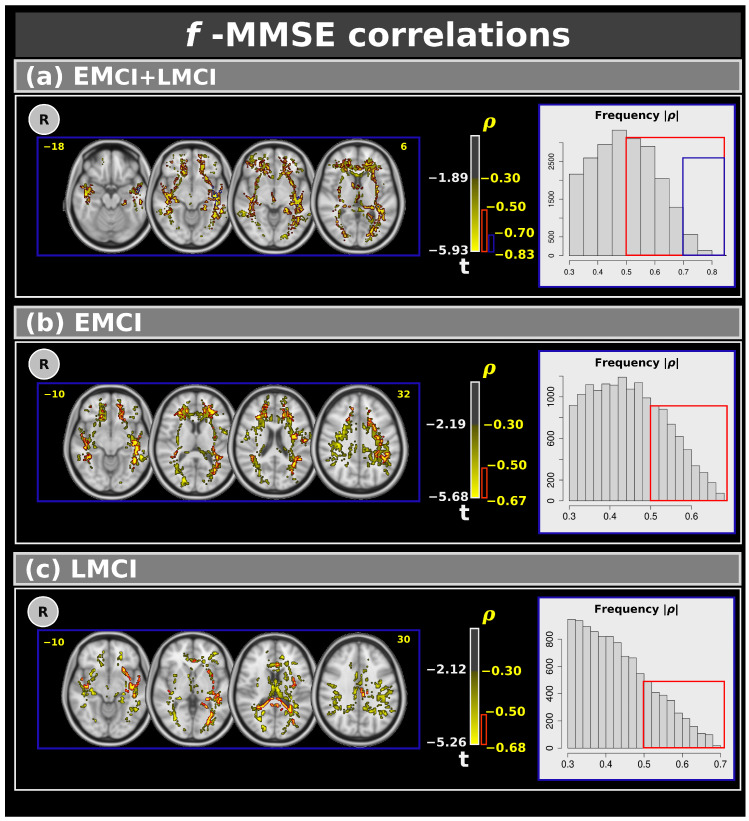
Correlations between the Mini–Mental State Examination and f-index for: (**a**) the combined EMCI + LMCI group, (**b**) only EMCI, and (**c**) only LMCI. The figure highlights clusters with effect sizes: >0.30 in yellow, >0.50 in red, and >0.70 in blue. The plots show the frequency. R: right. L: left. CN: cognitively normal. EMCI: Early Mild Cognitive Impairment. LMCI: Late Mild Cognitive Impairment. g: effect size for the post hoc comparisons. η^2^: effect size for the analysis of covariance (ANCOVA) model. vs: versus. The numbers in yellow are the MNI slices.In the red box the frequency of the ρ > 0.5. In the blue box the frequency of ρ > 0.7.

Figure 4 shows voxel-based correlations for the f-index with moderate, strong, and very strong effect sizes. Panel (a) presents correlations for the combined EMCI and LMCI groups. Clusters with moderate and strong effect sizes were found in several white matter regions, while clusters with very strong effect sizes were specifically located in the left inferior fronto-occipital fasciculus, left inferior longitudinal fasciculus, left retrolenticular part of the internal capsule, and left sagittal stratum. Panels (b) and (c) display the correlations between MMSE and EMCI (panel (b)) and LMCI (panel (c)). In these cases, only moderate and strong effect size correlations were observed. Table 5 shows the complete results for the f-index.

**Figure 5:**
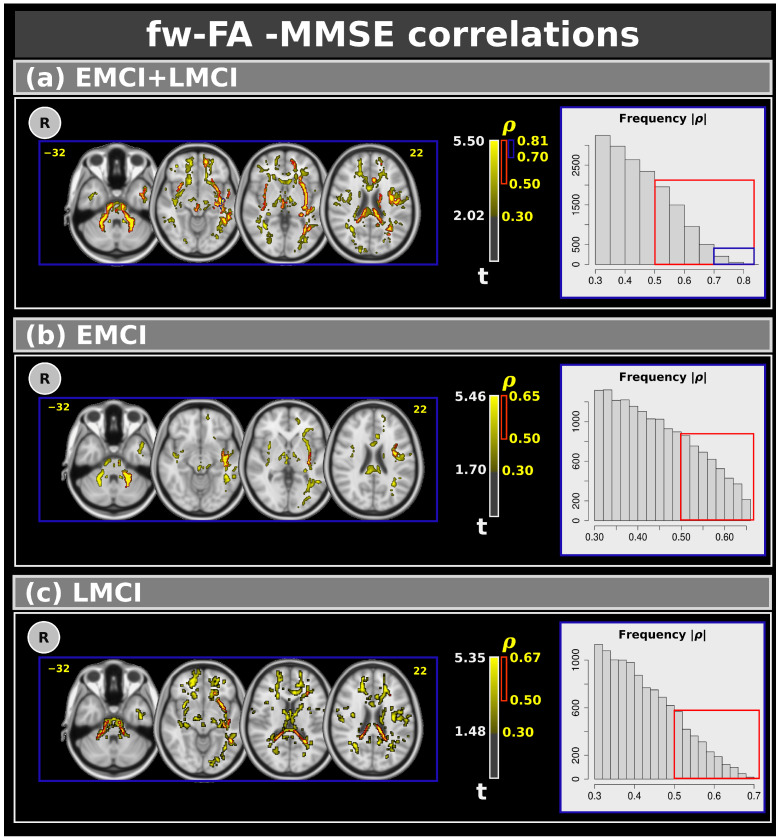
Correlations between the Mini–Mental State Examination and free-water fractional anisotropy metric for: (**a**) the combined EMCI + LMCI group, (**b**) only EMCI, and (**c**) only LMCI. The figure highlights clusters with effect sizes: > 0.30 in yellow, >0.50 in red, and >0.70 in blue. The plots show the frequency. R: right. L: left. CN: cognitively normal. EMCI: Early Mild Cognitive Impairment. LMCI: Late Mild Cognitive Impairment. g: effect size for the post hoc comparisons. η^2^: effect size for the analysis of covariance (ANCOVA) model. vs: versus. The numbers in yellow are the MNI slices. In the red box the frequency of the ρ > 0.5. In the blue box the frequency of ρ > 0.7.

Figure 5 shows voxel-based correlations for the fw-FA metric with moderate, strong, and very strong effect sizes. Panel (a) presents correlations for the combined EMCI and LMCI groups. Clusters with moderate and strong effect sizes were found in several white matter regions. Similarly to the f-index, we also identified clusters with very strong effect sizes in the left inferior fronto-occipital fasciculus, left inferior longitudinal fasciculus, and left sagittal stratum. Panels (b) and (c) display the correlations between MMSE and EMCI (panel (b)) and LMCI (panel (c)). Similarly to the f-index, only moderate and strong effect size correlations were observed. Table 6 shows the complete results for the fw-FA metric.

### 3.5. Region of Interest (ROI) Analysis on the White Matter

Table 7 shows the ROI analysis conducted for all fw-DTI metrics within the entire white matter. Significant group differences were identified in the f-index metric (CN vs. EMCI: t = −6.822, *p* < 0.001; CN vs. LMCI: t = −4.128, *p* < 0.001).

### 3.6. Volumetric Results

Table 8 presents the volumetric analysis of white matter, gray matter, and CSF volumes. Significant differences across groups were observed for all brain regions. For white matter, volume reductions were identified in the LMCI group, with significant differences between the CN and LMCI groups and between EMCI and LMCI. Regarding graygrey matter, significant reductions were observed in both the EMCI and LMCI groups compared to the CN group, with the MCI groups exhibiting decreased gray matter volumes. Lastly, CSF volumes were significantly increased in both the EMCI and LMCI groups compared to the CN group.

In summary, significant cognitive differences were found across groups, with MCI groups showing impaired performance in all cognitive tests compared to the CN groups. The f-index was higher in EMCI and LMCI groups, while fw-FA was lower compared to the CN groups. Volumetric analyses revealed reduced white and gray matter volumes and increased CSF volumes in the MCI groups. ROC analyses indicated fw-FA had the highest discriminative power for group classification. Correlations showed associations between the dMRI metrics and MMSE, with notable clusters in white matter regions.

## 4. Discussion

This study investigated changes in white matter microstructure in MCI and subsets of MCI using fw-DTI. We compared the fw index (f-index) and the fw-FA from fw-DTI in EMCI and LMCI groups against a CN group using data from the ADNI dataset [6]. Additionally, we examined voxel-based correlations between fw-DTI metrics and MMSE scores, aiming to examine the connection between neuroimaging data and cognitive function.

MMSE, GDS, CDR, and FAQ measures showed statistically significant differences among the three groups. Post hoc analyses revealed that all cognitive tests differentiated the CN group from both EMCI and LMCI, indicating early cognitive decline and depression in individuals with EMCI. However, only the FAQ measure revealed subtle differences between EMCI and LMCI. The FAQ score has previously been shown to identify general differences between the EMCI and LMCI groups [24], highlighting the impact of cognitive decline on daily functioning in the LMCI group.

The ANCOVA results (Figure 1) indicate that individuals with LMCI exhibit higher f-index values in significant clusters within the anterior thalamic radiation, forceps major, fornix, and left tapetum compared to CN individuals and those with EMCI. Notably, a significant cluster in the fornix, comprising about 10% of the entire fornix, underscores the importance of this white matter area in our findings. Post hoc analyses also revealed significant differences between CN and EMCI individuals, highlighting the subtlety of early cognitive changes. Additionally, several white matter areas showed significant differences between CN and LMCI individuals, with higher f-index values in the LMCI group, possibly indicating neurodegenerative changes leading to more sub-voxel free water.

The f-index measures the proportion of water molecules that diffuse freely within brain tissue, quantifying the amount of “unhindered” water as opposed to water restricted by the intricate structure of brain cells and fibers [17]. It could be a potential biomarker for various neurological diseases, such as AD [16,19,20], multiple sclerosis [35], and stroke [36,37], by revealing changes in the brain’s cellular organization and integrity. For instance, an increased f-index might indicate tissue damage or inflammation [19,38], while a decreased f-index might suggest the presence of dense fiber tracts [39].

Importantly, statistical differences in the f-index between EMCI and LMCI validate the progressive nature of cognitive decline within the MCI spectrum. The fornix and tapetum are notable regions where the LMCI group demonstrates higher f-index values compared to the EMCI group. These findings enhance our understanding of the dynamic changes occurring in white matter microstructure as cognitive impairment progresses.

In this study, we also analyzed the fw-FA metric, shown in Figure 2, and investigated differences in white matter microstructure under different cognitive states, including effect sizes to provide a more comprehensive understanding of the clinical implications of our findings [40]. More specifically, individuals with LMCI consistently exhibit lower fw-FA values compared to both the EMCI and CN groups. The identified clusters within the anterior thalamic radiation, forceps minor, external capsule, fornix, and right uncinate fasciculus demonstrate the widespread nature of these alterations. This result suggests a global disruption in the microstructural integrity of these white matter tracts in individuals with LMCI, potentially indicative of more advanced cognitive decline.

Additionally, we found significant differences between CN and EMCI individuals and between CN and LMCI individuals in several white matter areas. These findings indicate that individuals with EMCI and LMCI have lower fw-FA values than the CN group, indicative of compromised white matter microstructure. The most notable observation was the comparison between EMCI and LMCI, where fw-FA values were consistently lower in the LMCI group. This result suggests a progression of white matter microstructural abnormalities as cognitive impairment advances within the MCI spectrum.

One significant cluster, corresponding to differences in the f-index and fw-FA in the LMCI group, was observed in the fornix. The fornix plays a vital role in memory consolidation and retrieval by allowing the hippocampus to communicate with other brain regions, solidifying new memories, and ensuring their accessibility when needed [41]. Studies have suggested that the fornix might be one of the first structures in MCI and dementia to show signs of degeneration [42,43,44]; moreover, this degeneration can manifest as shrinkage in volume or as changes in its white matter microstructure, hindering its ability to efficiently conduct the vital flow of information [42,44]. Therefore, our findings indicate that the fornix appears particularly vulnerable in LMCI, showing more pronounced alterations than in EMCI, and might suggest a progressive decline in memory function as the condition worsens.

Other significant clusters of differences between the LMCI and CN groups were found in the corpus callosum, a thick band of white matter fibers connecting the left and right hemispheres of the brain. The corpus callosum is crucial in interhemispheric communication to support various cognitive functions [45,46,47]. Studies using MRI techniques have shown that individuals with MCI and dementia may exhibit reduced volume or altered microstructure in the splenium of the corpus callosum compared to CN cohorts [48,49,50,51]. Additionally, standard DTI has previously indicated disrupted white matter integrity in the corpus callosum of MCI patients, potentially impeding interhemispheric communication [52]. Thus, similar to the fornix, our findings suggest that alterations in the splenium may progress as MCI advances.

Other significant clusters for LMCI were found in the forceps major, minor, and tapetum. The forceps major is a posterior WM tract in the brain that connects the two occipital lobes. Functions attributed to the forceps major include visual processing, working memory, and attention [53], and thus damage to this tract in MCI may affect visual processing and memory consolidation. Studies have shown a reduced volume and altered microstructure of the forceps major in individuals with MCI compared to healthy adults, suggesting a potential breakdown in the white matter fibers here [54,55]. The tapetum is a thin layer of white matter fibers within the corpus callosum. It contributes to integrating sensorimotor information and is part of the larger neural network involved in cognitive processes [56].

Importantly, in this study, we also evaluated the effect sizes for all statistical analyses to quantify the magnitude of group differences. For ANCOVA, effect sizes were estimated using η^2^, with values of η^2^ ≥ 0.073 indicating a large effect. For post hoc group comparisons, effect sizes were calculated using g, with large effects defined as follows: CN vs. EMCI = |g| ≥ 0.29; CN vs. LMCI = |g| ≥ 0.41; EMCI vs. LMCI = |g| ≥ 0.47. Notably, large effect sizes were observed in several dMRI metrics, underscoring their potential clinical relevance. These findings suggest that the dMRI metrics could serve as robust indicators of neurodegenerative progression in MCI, providing valuable insights for early detection and monitoring.

The results of the ROC analysis provide valuable insights into the discriminative ability of MMSE, fw-FA, and the f-index in differentiating between the groups within significant clusters identified by fw-FA and the f-index. Across all comparisons, fw-FA demonstrated superior discriminative power, with the highest AUC values, particularly in the CN vs. LMCI (AUC = 0.81) and EMCI vs. LMCI (AUC = 0.84) comparisons. This suggests that fw-FA is a highly reliable biomarker for distinguishing between these groups. In contrast, the f-index exhibited moderate discriminative ability, particularly in the EMCI vs. LMCI comparison (AUC = 0.71), but its performance was consistently lower than fw-FA. MMSE showed the weakest performance for the EMCI vs. LMCI comparison, indicating that it may be less effective in detecting subtle group differences within MCI cohorts.

This study identified several negative voxel-based correlations (with effect sizes > 0.30 and >0.50) between the f-index and MMSE in both the EMCI and LMCI groups. Interestingly, for the combined EMCI and LMCI group, we found clusters with very strong effect sizes (>0.70) inside the left inferior fronto-occipital fasciculus, left inferior longitudinal fasciculus, left superior longitudinal fasciculus, left uncinate fasciculus, left retrolenticular part of internal capsule, and left sagittal stratum. The correlations were found predominantly in the left hemisphere of the brain, which is often associated with language and analytical processing [57]. Therefore, the findings might suggest that early disruptions in cognitive function are more pronounced in these areas, potentially leading to language and processing difficulties observed in conditions like AD. Additionally, the strong effect sizes (>0.70) in these regions suggest that these changes are robust and significant. Positive voxel-based correlations were found between fw-FA and MMSE scores. Similarly to the f-index, for only the combined EMCI and LMCI group, we found clusters with very strong effect sizes (>0.70) inside the left inferior fronto-occipital fasciculus, left inferior longitudinal fasciculus, and left sagittal stratum.

The volumetric analysis reveals significant structural changes across groups, consistent with the progression of cognitive impairment. The reduction in white matter volume in the LMCI group compared to the CN and EMCI groups suggests advanced white matter degeneration, likely due to axonal loss or demyelination. Similarly, decreased gray matter volume in both the EMCI and LMCI groups compared to the CN cohort highlights early cortical atrophy, which accelerates with disease progression. The increase in CSF volume in the EMCI and LMCI groups reflects compensatory changes due to brain atrophy.

Several studies have previously analyzed the EMCI and LMCI groups from the ADNI dataset. Jessen et al. conducted a comparative analysis of the risk of AD development across LMCI, EMCI, and subjective memory impairment (SMI) through cognitive testing, CSF, and neuroimaging biomarkers. They found that the LMCI group showed the greatest risk of developing AD, followed by the EMCI group and finally the SMI group [58]. In another study, Hua et al. found higher 24-month atrophy rates in the LMCI group (mean: 1.79%) compared to the EMCI group (mean: 1.04%) and CN group (mean: 0.47%), with regional variations in atrophy rates that were consistent with Alzheimer’s neurodegenerative patterns [59]. In a separate study, Zhang et al. investigated EMCI and LMCI using functional brain network metrics derived from resting-state functional MRI, finding differences in global efficiency, local efficiency, and the average clustering coefficient in the LMCI-EMCI comparison [60]. Additionally, Zheng et al. explored alterations in the complexity of resting-state brain blood oxygen level-dependent (BOLD) signals in patients with EMCI and LMCI, based on data from the ADNI database. The study included 345 participants and revealed a significant reduction in brain signal complexity, indicating increased regularity in the left fusiform gyrus for the EMCI group and in the rostral anterior cingulate cortex for the LMCI group [61]. However, it is essential to highlight that this study is the first investigation of differences in fw-DTI within the white matter microstructure between EMCI and LMCI individuals.

The clinical implications of these findings warrant further exploration. The identified fw-DTI biomarkers, f-index, and fw-FA could serve as valuable tools in clinical practice by facilitating discrimination of MCI subtypes and monitoring disease progression. Future research should focus on developing practical protocols for integrating fw-DTI into routine diagnostic workflows. For example, combining these imaging biomarkers with cognitive assessments or emerging fluid biomarkers could enhance the accuracy of MCI diagnosis and prognosis. Additionally, fw-DTI metrics could guide therapeutic decisions by identifying individuals who might benefit most from targeted interventions aimed at preserving white matter integrity. This integration could enable more personalized treatment approaches and improve patient outcomes in clinical settings.

There are some limitations to this study. The main limitation of the study is related to the single-shell dMRI acquisition. In order to maximize the sample size of EMCI and LMCI cohorts, we used only single-shell dMRI acquisitions available in the ADNI database. Although the single-shell fw-DTI approach has been used frequently, it has notable limitations that affect its accuracy and applicability. One limitation is the assumption that there is no exchange of water molecules between compartments; another limitation is that the tensor model does not account for the non-Gaussian nature of diffusion, which becomes more pronounced at higher b-values. Therefore, several spatial constraints have to be applied to this model [17,61], and the results from single-shell data should be carefully interpreted [18].

Single-shell dMRI data imposes limitations on the use of advanced diffusion models that require multi-shell acquisitions, such as diffusion kurtosis imaging (DKI) [50], neurite orientation dispersion and density imaging (NODDI) [62], and multi-shell free-water diffusion tensor imaging (fw-DTI) [61]. These techniques offer more detailed insights into tissue microstructure, providing valuable information about tissue complexity and microstructural changes beyond standard tensor-based methods. However, these advanced methods require multi-shell dMRI acquisitions, which were not available in the current dataset. Despite these limitations, fw-DTI using single-shell data remains a practical and valuable approach for investigating microstructural changes, especially in clinical settings where multi-shell acquisitions may not be feasible. Our findings demonstrate the clinical applicability of single-shell fw-DTI in distinguishing between early and late MCI, highlighting its utility even with these constraints. Future studies leveraging multi-shell data or advanced methods like DKI and NODDI could further validate and build upon the findings presented in this study.

Another limitation of this study is that the scanner and the number of directions were not constant across all participants in the ADNI study. We previously showed that group differences persisted across acquisitions with different scanners and the number of directions when assessed separately, suggesting that the differences observed relate to true biological differences rather than technical variability [63]. In the present study, ComBat harmonization was used to overcome this technical limitation and preserve the biological variability that underlies group differences [29]. Additionally, AD pathology may affect the ability to accurately differentiate CN individuals from those with EMCI. Variability in amyloid (A), tau (T), and neurodegeneration (N) markers among participants could result in overlapping characteristics between the CN and EMCI groups, potentially reducing the specificity of our findings.

A final limitation of this study could be associated with the EMCI and LMCI groups. ADNI categorizes these stages based solely on different levels of impairment in a single episodic memory measure used for MCI diagnosis, specifically one story from the Wechsler Memory Scale-Revised (WMS-R) Logical Memory II subtest. As the identification of these MCI subgroups is further refined, future studies should confirm the findings herein.

## 5. Conclusions

This study utilized fw-DTI to investigate changes in white matter integrity across the MCI spectrum. Additionally, we explored correlations between fw-DTI metrics and MMSE scores. Our findings indicate that fw-DTI is a promising and robust MRI analysis tool for distinguishing individuals with MCI from healthy controls and, importantly, for differentiating between EMCI and LMCI. The identified correlations suggest a potential link between alterations in white matter regions within the MCI population and cognitive decline. These findings highlight the potential of fw-DTI metrics as sensitive indicators for monitoring the progression of cognitive decline. Future research should focus on longitudinal studies to confirm the utility of fw-DTI in tracking disease progression over time. Additionally, clinical applications could explore integrating fw-DTI metrics into diagnostic workflows or intervention trials aimed at preserving white matter integrity. Developing tailored therapeutic strategies based on these microstructural changes may offer promising avenues to slow or prevent cognitive decline in individuals with MCI.

## Figures and Tables

**Table 1:** Complete subject characteristics: N: total number; F: females; CN: cognitively normal; EMCI: early MCI; LMCI: late MCI SD: standard deviation; MMSE: Mini–Mental State Examination; GDS: Geriatric Depression Scale; CDR: Clinical Dementia Rating; FAQ: Functional Activities Questionnaire; APOE: Apolipoprotein E. All the data represent mean ± standard deviation unless otherwise indicated. * with Bonferroni correction. ** from multi-shell.

		Included in the Final Analysis					
Group	N (#F)	N (#F)	Age (Year)	MMSE	GDS	FAQ	Global CDR
				[# Available]	[# Available]	[# Available]	[# Available]
CN	476 (284)	472 (282)	71.7 ± 8.3	29.07 ± 1.14 [471/472]	0.77 ± 1.13 [472/472]	0.15 ± 0.70 [429/472]	0.002 ± 0.033 [472/472]
EMCI	137 (55)	136 (54)	73.8 ± 7.7	28.27 ± 1.46 [136/136]	1.75 ± 1.61 [136/136]	1.81 ± 3.28 [129/136]	0.50 ± 0 [136/136]
LMCI	62 (28)	60 (26)	73.4 ± 5.9	27.48 ± 1.86 [60/60]	1.60 ± 1.36 [60/60]	4.81 ± 5.27 [58/60]	0.50 ± 0 [60/60]
Shapiro–Wilk			W = 0.989; *p* < 0.001	W = 0.815; *p* < 0.001	W = 0.772; *p* < 0.001	W = 0.407; *p* < 0.001	W = 0.573; *p* < 0.001
Kruskal–Wallis			χ^2^ = 5.03; *p* = 0.081	χ^2^ = 74.57; *p* < 0.001	χ^2^ = 66.58; *p* < 0.001	χ^2^ = 198.72; *p* < 0.001	χ^2^ = 651.68; *p* < 0.001
	Dunn test *	CN-EMCI		Z = 6.22; *p* < 0.001	Z = −7.18; *p* < 0.001	Z = −9.25; *p* < 0.001	Z = −22.24; *p* < 0.001
		CN-LMCI		Z = 6.91; *p* < 0.001	Z = −4.97; *p* < 0.001	Z = −12.02; *p* < 0.001	Z = −15.91; *p* < 0.001
		EMCI-LMCI		Z = 2.21; *p* = 0.082	Z = −0.11; *p* = 1.00	Z = −4.76; *p* < 0.001	Z = −0.10; *p* = 1.00
	Included in the final analysis						
ADNI diffusion MRI data	CN	EMCI	LMCI	Manufacturer			
30 dir + 1 b0	74	11	0	SIEMENS			
32 dir + 4 b0	24	5	5	GE MEDICAL SYSTEMS			
41 dir + 5 b0	61	84	37	GE MEDICAL SYSTEMS			
48 dir + 6 b0	59	0	0	GE MEDICAL SYSTEMS			
48 dir + 2 b0 **	87	11	3	SIEMENS			
54 dir + 2 b0	167	25	15	SIEMENS			
Motion/Outliers	N (#F)	Motion (ABS)	Motion (REL)	Outliers (TOT)			
CN	472 (282)	0.98 ± 0.56	0.20 ± 0.12	0.07 ± 0.04			
EMCI	136 (54)	0.96 ± 0.58	0.20 ± 0.13	0.08 ± 0.05			
LMCI	60 (26)	1.03 ± 0.53	0.21 ± 0.11	0.09 ± 0.05			
Shapiro–Wilk		W = 0.959; *p* < 0.001	W = 0.948; *p* < 0.001	W = 0.941; *p* < 0.001			
Kruskal–Wallis		χ^2^ = 1.161; *p* = 0.561	χ^2^ = 0.136 *p* = 0.934	χ^2^ = 3.226 *p* = 0.200			
	Included in the Final Analysis						
APOE	CN (#424)	EMCI (#129)	LMCI (#57)				
E2 E2	46 (10.85%)	1 (0.78%)	0 (0%)				
E2 E3	0 (0%)	12 (9.30%)	3 (5.26%)				
E2 E4	6 (1.42%)	3 (2.33%)	3 (5.26%)				
E3 E3	239 (56.37%)	66 (51.16%)	16 (28.07%)				
E3 E4	116 (27.36%)	37 (28.68%)	29 (50.88%)				
E4 E4	17 (4.01%)	10 (7.75%)	6 (10.53%)				

**Table 2:** Complete MRI scanners and dMRI acquisition information: ms: milliseconds. TR: repetition time—measured in milliseconds (ms). TE: echo time—measured in milliseconds (ms). mm^3^: cubic millimeters. mm^2^: Square Millimeters. N: number of images without diffusion. s: seconds. dir: number of diffusion directions. * from multi-shell—only used b = 1000 with 48 directions. ** Data from multi-shell dMRI.

Diffusion MRI	SIEMENS 30 dir + 1 b0	GE 32 dir + 4 b0	GE 41 dir + 5 b0	GE 48 dir + 6 b0	SIEMENS 48 dir + 2 b0 *	SIEMENS 54 dir + 2 b0
Manufacturer	Siemens	GE	GE	GE	Siemens	Siemens
Model	Verio	DISCOVERY MR750	DISCOVERY MR750	DISCOVERY MR750	Prisma Fit	Prisma Fit
Repetition time (TR) (ms)	12,400	15,354	9050	7800	3400	7200
Echo time (TE) (ms)	95	74.6	62.8	60.9	71	56
Voxel size (mm^3^)	2.0 × 2.0 × 2.0	0.9 × 0.9 × 2.0	1.4 × 1.4 × 2.7	0.9 × 0.9 × 2.0	2.0 × 2.0 × 2.0	2.0 × 2.0 × 2.0
Flip-angle	90°	90°	90°	90°	90°	90°
N directions	30	32	41	48	126 **	54
N b0 images	1	4	5	6	2	2
b value (s/mm^2^)	1000	1000	1000	1000	500, 1000, 2000 **	1000

**Table 3:** The outcomes of the analysis of covariance (ANCOVA) (at a False Discovery Rate (FDR) < 0.05), along with post hoc comparisons, for the f-index (corresponding to Figure 2). % Vol: the volume of the significant cluster within the relative white matter region. <t> the mean of the t-value inside the significant clusters. η2 and g are the effect sizes for the ANCOVA model and the post hoc comparisons, respectively. JHU: Johns Hopkins University white matter atlas. WM: white matter. max: maximum. CN: cognitively normal. EMCI: Early Mild Cognitive Impairment. LMCI: Late Mild Cognitive Impairment. L: left. R: right. ICBM: International Consortium for Brain Mapping, the standardized brain templates and atlases used in neuroimaging. DTI: diffusion tensor imaging. Fornix (cres): crescent-shaped part of the fornix.

	ANCOVA			POST-HOC COMPARISONS								
				CN < EMCI			CN < LMCI			EMCI < LMCI		
JHU WM Tractography	% Vol	<F>	max η^2^	% Vol	<t>	max |g|	% Vol	<t>	max |g|	% Vol	<t>	max |g|
Anterior thalamic radiation L	13.2	6.207	0.954	7.24	−2.043	−0.34	14.11	−2.489	−0.665	2.07	−2.205	−1.022
Anterior thalamic radiation R	9.72	5.018	0.484	7.09	−2.298	−0.322	9.75	−2.319	−0.637	1.55	−2.752	−0.838
Cortical spinal tract L	10.29	4.294	0.113	17.14	−2.481	−0.364	-	-	-	-	-	-
Cortical spinal tract R	4.49	4.17	0.114	6.72	−2.445	−0.338	-	-	-	-	-	-
Cingulum cingulate gyrus L	10.89	4.518	0.147	5.81	−2.044	−0.33	-	-	-	-	-	-
Cingulum cingulate gyrus R	6.02	3.954	0.12	-	-	-	-	-	-	-	-	-
Forceps major	6.87	4.25	0.128	-	-	-	13.13	−2.092	−0.555	1.06	−2.364	−0.643
Forceps minor	7.15	4.781	0.387	5.34	−1.937	−0.371	6.37	−2.474	−0.582	-	-	-
Inferior fronto-occipital fasc L	7.76	5.242	0.192	9.64	−2.124	−0.369	10.95	−2.195	−0.673	-	-	-
Inferior fronto-occipital fasc R	6	4.759	0.176	2.34	−2.262	−0.324	9.25	−2.381	−0.639	-	-	-
Inferior longitudinal fasc L	11.72	4.583	0.19	11.77	−2.143	−0.384	13.12	−2.43	−0.586	-	-	-
Inferior longitudinal fasc R	6.86	4.76	0.179	-	-	-	11.21	−2.106	−0.664	-	-	-
Superior longitudinal fasc L	19.53	4.345	0.187	22.41	−2.297	−0.484	9.31	−2.073	−0.517	-	-	-
Superior longitudinal fasc R	9.29	3.933	0.155	12.41	−2.264	−0.387	-	-	-	-	-	-
Uncinate fasc L	3.03	5.871	0.189	10.33	−2.02	−0.296	7.91	−2.217	−0.664	-	-	-
Sup longitudinal Fasc temporal L	24.06	4.441	0.187	27.23	−2.368	−0.484	12.88	−2.024	−0.507	-	-	-
Sup longitudinal fasc temporal R	14.56	3.953	0.138	18.7	−2.332	−0.373	-	-	-	-	-	-
**ICBM-DTI 81**	**% Vol**	**<F>**	**max η^2^**	**% Vol**	**<t>**	**max |g|**	**% Vol**	**<t>**	**max |g|**	**% Vol**	**<t>**	**max |g|**
Genu of corpus callosum	22.63	4.402	0.17	11.05	−1.924	−0.348	21.74	−2.314	−0.582	-	-	-
Body of corpus callosum	13.66	4.075	0.128	7.88	−2.123	−0.361	11.18	−2.214	−0.514	-	-	-
Splenium of corpus callosum	8.23	3.638	0.144	-	-	-	11.43	−2.639	−0.55	-	-	-
Fornix	57.21	7.779	0.242	-	-	-	63.58	−3.167	−0.735	9.26	−2.328	−0.521
Anterior limb of internal capsule L	1.26	2.865	0.076	1.36	−1.978	−0.307	1.09	−1.48	−0.355	-	-	-
Posterior limb of internal capsule L	13.41	3.648	0.092	20.39	−2.227	−0.326	-	-	-	-	-	-
Retrolenticular part of internal capsule L	22.76	4.653	0.144	27.91	−2.325	−0.414	-	-	-	-	-	-
Anterior corona radiata R	4.37	5.005	0.115	26.54	−2.4	−0.326	-	-	-	-	-	-
Anterior corona radiata L	1.44	3.666	0.081	15.91	−2.086	−0.318	-	-	-	-	-	-
Superior corona radiata R	16.48	3.988	0.109	30.83	−2.377	−0.348	-	-	-	-	-	-
Superior corona radiata L	39.22	4.452	0.13	62.48	−2.543	−0.368	-	-	-	-	-	-
Posterior corona radiata R	10.97	4.172	0.114	8.96	−2.318	−0.337	-	-	-	-	-	-
Posterior corona radiata L	18.12	4.024	0.138	30.64	−2.257	−0.371	-	-	-			
Posterior thalamic radiation R	19.31	4.172	0.161	-	-	-	22.89	−2.533	−0.563	-	-	-
Posterior thalamic radiation L	18.05	5.111	0.19	-	-	-	24.81	−2.548	−0.586	-	-	-
Sagittal stratum R	24.73	5.05	0.176	-	-	-	27.74	−2.239	−0.597	-	-	-
Sagittal stratum L	22.9	4.108	0.147	20.57	−1.959	−0.315	25.64	−2.346	−0.559	-	-	-
External capsule L	11.67	4.391	0.134	22.89	−2.139	−0.357	12.1	−2.393	−0.507	-	-	-
Cingulum (cingulate gyrus) R	10.42	3.55	0.088	-	-	-	-	-	-	-	-	-
Cingulum (cingulate gyrus) L	17.23	4.374	0.147	15.59	−2.105	−0.326	-	-	-	-	-	-
Fornix (cres)/Stria terminalis R	56.58	7.441	0.211	-	-	-	60.14	−3.103	−0.698	-	-	-
Fornix (cres)/Stria terminalis L	30.22	6.762	0.217	14.13	−2.204	−0.406	40	−2.493	−0.604	-	-	-
Superior longitudinal fasciculus R	21.49	3.912	0.124	31.18	−2.29	−0.348	-	-	-	-	-	-
Superior longitudinal fasciculus L	39.74	4.464	0.187	50.17	−2.417	−0.484	-	-	-	-	-	-
Superior fronto-occipital fasciculus R	0.79	3.554	0.04	14.79	−1.843	−0.344	-	-	-	-	-	-
Superior fronto-occipital fasciculus L	13.41	3.562	0.084	24.26	−1.92	−0.311	-	-	-	-	-	-
Uncinate fasciculus L	18.88	3.334	0.115	22.87	−1.842	−0.308	-	-	-	-	-	-
Tapetum R	71.81	4.716	0.122	-	-	-	79.7	−2.596	−0.476	-	-	-
Tapetum L	73.33	6.838	0.153	-	-	-	74	−3.188	−0.626	37.17	−2.581	−0.652

**Table 4:** The outcomes of the analysis of covariance (ANCOVA) (at a False Discovery Rate (FDR) < 0.05), along with post hoc comparisons, for free-water fractional anisotropy (corresponding to Figure 3). % Vol: the volume of the significant cluster within the relative white matter region. <t> the mean of the t-value inside the significant clusters. η^2^ and g are the effect sizes for the ANCOVA model and the post hoc comparisons, respectively. JHU: Johns Hopkins University white matter atlas. WM: white matter. max: maximum. CN: cognitively normal. EMCI: Early Mild Cognitive Impairment. LMCI: Late Mild Cognitive Impairment. L: left. R: right. ICBM: International Consortium for Brain Mapping, the standardized brain templates and atlases used in neuroimaging. DTI: diffusion tensor imaging. Fornix (cres): crescent-shaped part of the Fornix.

	ANCOVA			POST-HOC COMPARISONS								
				CN > EMCI			CN > LMCI			EMCI > LMCI		
JHU WM Tractography	% Vol	<F>	max η^2^	% Vol	<t>	max |g|	% Vol	<t>	max |g|	% Vol	<t>	max |g|
Anterior thalamic radiation L	30.94	7.777	0.099	13.09	2.96	0.678	20.26	3.147	0.881	0.74	2.129	0.559
Anterior thalamic radiation R	26.42	7.979	0.089	11.36	3.103	0.607	19.3	2.974	0.877	0.2	2.08	0.598
Cortical spinal tract L	21.18	6.747	0.087	11.55	3.068	0.605	8.01	2.649	0.606	-	-	-
Cortical spinal tract R	19.25	5.483	0.089	6.37	2.514	0.628	10.27	2.364	0.811	0.21	2.471	0.598
Cingulum cingulate gyrus L	70.62	7.626	0.07	52.97	2.952	0.571	54.79	3.049	0.76	-	-	-
Cingulum cingulate gyrus R	65.6	6.995	0.064	36.6	2.826	0.569	53.61	2.945	0.694	-	-	-
Cingulum hippo L	47.6	10.68	0.088	-	-	-	40.62	3.353	0.871	-	-	-
Forceps major	21.24	4.447	0.043	-	-	-	7.46	2.503	0.6	-	-	-
Forceps minor	33.76	5.561	0.051	12.22	2.844	0.471	23.83	2.752	0.672	0.28	2.079	0.519
Inferior fronto-occipital fasc L	33.17	7.254	0.075	15.75	3.302	0.598	22.62	3.03	0.788	0.24	2.107	0.609
Inferior fronto-occipital fasc R	28.89	5.655	0.056	8.42	2.853	0.483	15.91	2.754	0.671	-	-	-
Inferior longitudinal fasc L	35.65	6.725	0.117	19.21	3.134	0.61	21.05	2.916	1.037	0.14	2.07	0.513
Inferior longitudinal fasc R	29.86	5.847	0.053	13.97	2.844	0.457	17.45	2.746	0.666	-	-	-
Superior longitudinal fasc L	38.63	5.816	0.062	22.87	2.831	0.598	23.24	2.792	0.65	-	-	-
Superior longitudinal fasc R	23.36	4.353	0.044	9.53	2.535	0.392	9.66	2.528	0.607	-	-	-
Uncinate fasc L	33.78	7.32	0.088	16.94	3.133	0.558	28.14	3.02	0.852	-	-	-
Uncinate fasc R	33.56	6.006	0.046	4.67	2.805	0.409	22.64	3.045	0.693	0.98	1.95	0.566
Sup longitudinal fasc temporal L	50.39	6.428	0.062	32.04	2.989	0.598	32.68	2.881	0.65	-	-	-
Sup longitudinal fasc temporal R	32.27	4.596	0.044	14.66	2.625	0.392	16.31	2.578	0.579	-	-	-
**ICBM-DTI 81**	**% Vol**	**<F>**	**max η^2^**	**% Vol**	**<t>**	**max |g|**	**% Vol**	**<t>**	**max |g|**	**% Vol**	**<t>**	**max |g|**
Middle cerebellar peduncle	35.79	8.198	0.123	20.17	2.698	0.761	10.02	1.599	0.785	-	-	-
Pontine crossing tract (a part of MCP)	39.8	4.614	0.025	-	-	-	-	-	-	-	-	-
Genu of corpus callosum	51.18	6.126	0.049	35.44	2.912	0.471	29.88	2.698	0.642	-	-	-
Body of corpus callosum	77.49	10.53	0.085	68.53	3.526	0.638	65.98	3.283	0.83	-	-	-
Splenium of corpus callosum	47.19	6.115	0.056	22.08	2.845	0.488	32.72	2.895	0.716	-	-	-
Fornix (column and body of fornix)	69.35	18.3	0.119	59.18	4.014	0.652	69.35	4.329	1.04	8.69	2.784	0.723
Corticospinal tract R	33.63	4.919	0.056	12.7	2.842	0.423	8.22	2.585	0.639	-	-	-
Corticospinal tract L	12.26	5.545	0.04	4.16	2.741	0.344	-	-	-	-	-	-
Medial lemniscus R	29.86	5.382	0.052	-	-	-	-	-	-	-	-	-
Medial lemniscus L	32.47	4.804	0.033	-	-	-	-	-	-	-	-	-
Inferior cerebellar peduncle R	52.17	11.78	0.116	28.72	3.725	0.691	42.56	2.507	0.951	-	-	-
Inferior cerebellar peduncle L	44.01	12.65	0.139	25.31	3.466	0.773	-	-	-	-	-	-
Superior cerebellar peduncle R	46.77	7.564	0.078	20.46	2.606	0.524	34.17	2.405	0.784	-	-	-
Superior cerebellar peduncle L	55.54	7.851	0.08	36.79	2.696	0.607	-	-	-	-	-	-
Cerebral peduncle R	35.82	4.859	0.036	-	-	-	29.89	2.812	0.58	-	-	-
Cerebral peduncle L	21.55	4.099	0.035	-	-	-	10.76	2.465	0.463	-	-	-
Anterior limb of internal capsule L	10.47	3.459	0.031	3.38	2.228	0.266	-	-	-	-	-	-
Posterior limb of internal capsule R	10.31	4.049	0.024	-	-	-	5.67	2.413	0.417	-	-	-
Posterior limb of internal capsule L	17.99	4.934	0.04	9.12	2.632	0.322	8.66	2.419	0.447	-	-	-
Retrolenticular part of internal capsule R	49.74	6.096	0.044	36.9	2.945	0.42	19.68	2.763	0.668	-	-	-
Retrolenticular part of internal capsule L	66.42	9.5	0.065	57.63	3.367	0.5	53.79	3.112	0.707	-	-	-
Anterior corona radiata R	36.11	4.725	0.047	-	-	-	20.79	2.849	0.674	-	-	-
Anterior corona radiata L	23.02	5.076	0.049	7.68	2.79	0.427	15.95	2.652	0.672	-	-	-
Superior corona radiata R	27.96	5.953	0.053	14.28	3.051	0.502	14.77	2.807	0.583	-	-	-
Superior corona radiata L	33.59	8.964	0.107	21.54	3.787	0.688	12.23	3.66	0.878	-	-	-
Posterior corona radiata R	21.19	6.484	0.044	13.01	3.173	0.457	11.91	2.551	0.572	-	-	-
Posterior corona radiata L	30.75	7.607	0.075	18.04	3.496	0.629	17.88	2.719	0.63	-	-	-
Posterior thalamic radiation R	41.52	4.876	0.052	10.05	2.847	0.483	21.42	2.36	0.571	-	-	-
Posterior thalamic radiation L	45.45	4.9	0.053	6.94	3.142	0.516	17.17	2.412	0.539	-	-	-
Sagittal stratum R	43.27	5.997	0.044	25.72	2.625	0.423	30.39	2.592	0.587	-	-	-
Sagittal stratum L	44.29	8.269	0.065	37.29	3.148	0.517	31.91	3.05	0.672	-	-	-
External capsule R	51.54	6.707	0.045	7.91	2.774	0.389	39.44	3.01	0.686	1.59	2.117	0.524
External capsule L	61.95	8.434	0.067	43.98	2.826	0.52	55.93	3.251	0.854	2.83	2.655	0.712
Cingulum (cingulate gyrus) R	71.9	8.436	0.062	55.04	2.948	0.56	63.45	3.182	0.694	-	-	-
Cingulum (cingulate gyrus) L	76.12	8.295	0.069	63.54	3.054	0.543	62.09	3.092	0.669	-	-	-
Cingulum (hippocampus) L	75.5	12.48	0.088	-	-	-	73.51	3.602	0.871	-	-	-
Fornix (cres)/stria terminalis R	73.4	10.77	0.087	55.16	3.071	0.518	68.77	3.367	0.931	2.94	2.345	0.637
Fornix (cres)/stria terminalis L	73.6	18.74	0.118	70.93	4.259	0.682	73.6	4.292	0.97	-	-	-
Superior longitudinal fasciculus R	45.48	4.486	0.044	21.99	2.589	0.52	17.69	2.556	0.579	-	-	-
Superior longitudinal fasciculus L	62.32	6.214	0.047	45.13	2.942	0.491	34.94	2.754	0.617	-	-	-
Superior fronto-occipital fasciculus L	30.77	4.698	0.03	-	-	-	24.65	2.202	0.437	-	-	-
Uncinate fasciculus R	86.58	5.978	0.037	-	-	-	77.63	3.056	0.657	3.68	1.743	0.513
Uncinate fasciculus L	63.83	10.05	0.051	60.64	3.196	0.472	56.38	3.177	0.623	-	-	-
Tapetum R	70.81	5.744	0.031	17.62	2.454	0.252	67.28	2.794	0.506	-	-	-
Tapetum L	68.67	9.624	0.063	-	-	-	66.83	3.372	0.765	-	-	-

**Table 5:** The outcomes of the correlations between the f-index and Mini–Mental State Examination for different effect size thresholds. % Vol: the volume of the significant cluster within the relative white matter region. <ρ>: the mean of the effect size value inside the significant clusters. JHU: Johns Hopkins University white matter atlas. WM: white matter. max: maximum. CN: cognitively normal. EMCI: Early Mild Cognitive Impairment. LMCI: Late Mild Cognitive Impairment. L: left. R: right. ICBM: International Consortium for Brain Mapping, the standardized brain templates and atlases used in neuroimaging. DTI: diffusion tensor imaging. Fornix (cres): the crescent-shaped part of the fornix.

	EMCI + LMCI						EMCI				LMCI			
	Moderate Effect		Strong Effect		Very Strong Effect		Moderate Effect		Strong Effect		Moderate Effect		Strong Effect	
JHU WM Tractography	% Vol	<ρ>	% Vol	<ρ>	% Vol	<ρ>	% Vol	<ρ>	% Vol	<ρ>	% Vol	<ρ>	% Vol	<ρ>
Anterior thalamic radiation L	36.14	−0.42	19.31	−0.57	-	-	25.15	−0.44	8.49	−0.54	20.03	−0.4	1.75	0.54
Anterior thalamic radiation R	37.18	−0.4	14.2	−0.52	-	-	25.23	−0.45	7.8	−0.53	8.34	−0.38	-	-
Cortical spinal tract L	10.33	−0.35	2.14	−0.56	-	-	9.97	−0.42	1.56	−0.54	6.61	−0.37	-	-
Cingulum cingulate gyrus L	43.89	−0.4	19.01	−0.54	-	-	34.26	−0.42	8.53	−0.51	21.53	−0.39	2	0.55
Cingulum cingulate gyrus R	40.64	−0.38	17.15	−0.51	-	-	24.78	−0.38	4.16	−0.48	21.82	−0.39	-	-
Cingulum hippo L	39.17	−0.42	27.38	−0.58	-	-	-	-	-	-	28.48	−0.4	1.87	0.54
Forceps major	31.1	−0.4	13.15	−0.58	-	-	17.79	−0.38	-	-	20.42	−0.44	5.5	0.59
Forceps minor	39.85	−0.4	14.6	−0.54	-	-	33.46	−0.42	8.44	−0.53	11.41	−0.42	0.82	0.64
Inferior fronto-occipital fasc L	51.33	−0.46	32.83	−0.61	1.47	−0.79	44.18	−0.45	19.35	−0.53	27.48	−0.43	6.36	0.6
Inferior fronto-occipital fasc R	44.27	−0.4	19.53	−0.54	-	-	36.78	−0.42	8.95	−0.5	14.42	−0.4	0.73	0.59
Inferior longitudinal fasc L	42.96	−0.47	30.54	−0.63	2.25	−0.79	36.37	−0.45	16.66	−0.53	28.89	−0.43	6.37	0.59
Inferior longitudinal fasc R	42.23	−0.42	25.27	−0.59	-	-	36.94	−0.41	9.5	−0.5	22.18	−0.4	1.5	0.55
Superior longitudinal fasc L	43.93	−0.42	23.91	−0.59	0.32	−0.78	36.32	−0.44	14.74	−0.51	24.19	−0.41	1.66	0.6
Superior longitudinal fasc R	28.09	−0.36	8.67	−0.52	-	-	24.22	−0.39	-	-	10.75	−0.37	-	-
Uncinate fasc L	47.56	−0.44	26.51	−0.56	0.59	−0.76	39.48	−0.45	15.19	−0.52	21.78	−0.42	3.58	0.58
Uncinate fasc R	44.64	−0.43	22.72	−0.56	-	-	36.87	−0.43	7.85	−0.53	18.58	−0.4	-	-
Sup longitudinal fasc temporal L	48.95	−0.45	31.48	−0.61	0.76	−0.78	43.23	−0.45	20.76	−0.51	28.22	−0.41	1.9	0.58
Sup longitudinal fasc temporal R	35.97	−0.38	11.78	−0.53	-	-	34.9	−0.41	-	-	13.18	−0.37	-	-
**ICBM-DTI 81**	**% Vol**	**<ρ>**	**% Vol**	**<ρ>**	**% Vol**	**<ρ>**	**% Vol**	**<ρ>**	**% Vol**	**<ρ>**	**% Vol**	**<ρ>**	**% Vol**	**<ρ>**
Genu of corpus callosum	49.98	−0.36	8.89	−0.59	-	-	36.52	−0.37	4.25	−0.64	17.06	−0.37	0.66	−0.58
Body of corpus callosum	30.2	−0.31	4.14	−0.55	-	-	11.3	−0.35	0.39	−0.62	19.44	−0.39	3.84	−0.52
Splenium of corpus callosum	26.36	−0.37	13.56	−0.63	-	-	7.57	−0.31	-	-	23.54	−0.46	14.68	−0.56
Fornix (column and body of fornix)	64.19	−0.44	40.21	−0.68	-	-	-	-	-	-	62.06	−0.44	-	-
Retrolenticular part of internal capsule R	17.65	−0.33	5.25	−0.56	-	-	19.72	−0.36	2.62	−0.58	1.83	−0.3	-	-
Retrolenticular part of internal capsule L	40.58	−0.45	24.58	−0.73	1.46	−0.72	34.26	−0.48	15.92	−0.76	29.16	−0.39	1.7	−0.54
Anterior corona radiata R	71.48	−0.42	22.82	−0.61	-	-	75.38	−0.47	16.88	−0.7	2.48	−0.36	-	-
Anterior corona radiata L	76.62	−0.45	34	−0.67	-	-	71.75	−0.49	25.06	−0.7	26.08	−0.4	-	-
Superior corona radiata R	24.89	−0.34	-	-	-	-	42.84	−0.39	-	-	0.48	−0.36	-	-
Superior corona radiata L	46.46	−0.35	7.54	−0.57	-	-	46.5	−0.42	6.53	−0.68	19.66	−0.39	0.61	−0.6
Posterior corona radiata R	23.66	−0.33	8.99	−0.57	-	-	20.25	−0.33	-	-	7.99	−0.39	-	-
Posterior corona radiata L	40.79	−0.35	11.95	−0.56	-	-	36.86	−0.36	2.67	−0.62	22.78	−0.37	-	-
Posterior thalamic radiation R	46.48	−0.38	17.4	−0.6	-	-	31.92	−0.39	0.15	−0.68	25.35	−0.39	-	-
Posterior thalamic radiation L	53.27	−0.47	36.15	−0.71	-	-	41.05	−0.43	11.71	−0.65	34.26	−0.46	9.48	−0.6
Sagittal stratum R	58.3	−0.41	36.76	−0.63	-	-	57	−0.39	16.56	−0.61	25.94	−0.33	0.72	−0.52
Sagittal stratum L	50.69	−0.46	39.89	−0.71	4.26	−0.79	48.81	−0.46	30.39	−0.65	25.68	−0.36	5.47	−0.53
External capsule R	44.22	−0.36	14.92	−0.57	-	-	34.17	−0.37	1.82	−0.6	20.32	−0.38	-	-
External capsule L	50.1	−0.41	23.02	−0.63	-	-	34.04	−0.39	4.46	−0.64	33.42	−0.42	8.7	−0.58
Cingulum (cingulate gyrus) R	34.2	−0.36	12.85	−0.55	-	-	23.95	−0.37	-	-	18.66	−0.36	-	-
Cingulum (cingulate gyrus) L	36.64	−0.37	16.65	−0.59	-	-	25.81	−0.38	-	-	23.63	−0.36	3.78	−0.52
Cingulum (hippocampus) L	34.81	−0.41	24.85	−0.63	-	-	-	-	-	-	25.11	−0.39	-	-
Fornix (cres)/stria terminalis R	63.79	−0.46	52.4	−0.68	-	-	42.26	−0.34	-	-	53.91	−0.42	-	-
Fornix (cres)/stria terminalis L	47.64	−0.43	40.27	−0.63	-	-	43.64	−0.35	-	-	32.8	−0.38	12.62	−0.59
Superior longitudinal fasciculus R	49.19	−0.38	17.44	−0.62	-	-	49.7	−0.41	2.21	−0.6	19.74	−0.37	-	-
Superior longitudinal fasciculus L	67.84	−0.45	40.82	−0.7	-	-	61.15	−0.46	28.69	−0.67	40.53	−0.41	1.7	−0.62
Superior fronto-occipital fasciculus R	42.21	−0.33	-	-	-	-	45.96	−0.39	-	-	0	0	-	-
Superior fronto-occipital fasciculus L	45.36	−0.32	-	-	-	-	15.78	−0.32	-	-	31.95	−0.35	-	-
Uncinate fasciculus R	27.89	−0.35	9.47	−0.59	-	-	15.79	−0.33	-	-	27.89	−0.35	-	-
Tapetum R	85.91	−0.42	35.57	−0.54	-	-	5.54	−0.37	-	-	74.33	−0.48	25.84	−0.61
Tapetum L	74	−0.48	65.33	−0.7	-	-	20.83	−0.33	-	-	71.83	−0.53	47.83	−0.61

**Table 6:** The outcomes of the correlations between free-water-fractional anisotropy and Mini–Mental State Examination for different effect size thresholds. % Vol: the volume of the significant cluster within the relative white matter region. <ρ>: the mean of the effect size value inside the significant clusters. JHU: Johns Hopkins University white matter atlas. WM: white matter. max: maximum. CN: cognitively normal. EMCI: Early Mild Cognitive Impairment. LMCI: Late Mild Cognitive Impairment. L: left. R: right. ICBM: International Consortium for Brain Mapping, the standardized brain templates and atlases used in neuroimaging. DTI: diffusion tensor imaging. Fornix (cres): the crescent-shaped part of the fornix.

	EMCI + LMCI						EMCI				LMCI			
	Moderate Effect		Strong Effect		Very Strong Effect		Moderate Effect		Strong Effect		Moderate Effect		Strong Effect	
JHU WM Tractography	% Vol	<ρ>	% Vol	<ρ>	% Vol	<ρ>	% Vol	<ρ>	% Vol	<ρ>	% Vol	<ρ>	% Vol	<ρ>
Anterior thalamic radiation L	32.41	0.39	8.12	0.55	-	-	13.49	0.37	-	-	23.74	0.37	2.64	0.56
Anterior thalamic radiation R	26.2	0.39	6.68	0.56	-	-	7.83	0.39	-	-	23.21	0.38	-	-
Cortical spinal tract L	19.75	0.42	5.6	0.6	-	-	11.81	0.41	2.88	0.65	14.46	0.38	3.06	0.55
Cortical spinal tract R	15.69	0.38	2.48	0.59	-	-	6.77	0.39	-	-	10.57	0.35	0.78	0.53
Cingulum cingulate gyrus L	42.59	0.41	13.44	0.56	-	-	19.28	0.39	-	-	29.26	0.37	-	-
Cingulum cingulate gyrus R	42.53	0.37	-	-	-	-	15.92	0.37	-	-	16.76	0.34	-	-
Forceps major	17.48	0.37	2.65	0.56	-	-	3.22	0.37	-	-	11.53	0.38	2.23	0.54
Forceps minor	29.69	0.38	3.2	0.56	-	-	4.13	0.38	-	-	23.95	0.38	-	-
Inferior fronto-occipital fasc L	42.73	0.43	14.85	0.6	0.53	0.76	24.73	0.43	3.87	0.63	29.15	0.38	4.85	0.56
Inferior fronto-occipital fasc R	24.4	0.36	1.44	0.52	-	-	3.8	0.35	-	-	14.48	0.36	-	-
Inferior longitudinal fasc L	39.11	0.42	12.96	0.58	0.78	0.77	27.32	0.42	3.59	0.64	20.06	0.36	2.3	0.52
Inferior longitudinal fasc R	18.73	0.36	-	-	-	-	4.93	0.36	-	-	3.44	0.32	-	-
Superior longitudinal fasc L	33.41	0.4	8.09	0.58	-	-	19.23	0.4	2.27	0.61	20.05	0.37	1.24	0.6
Superior longitudinal fasc R	20.61	0.35	0.26	0.54	-	-	5.66	0.37	-	-	11.22	0.33	-	-
Uncinate fasc L	41.51	0.43	12.59	0.61	-	-	19.81	0.42	1	0.54	28.74	0.41	8.38	0.57
Uncinate fasc R	27.58	0.38	2.73	0.58	-	-	4.44	0.36	-	-	18.63	0.41	-	-
Sup longitudinal fasc temporal L	42.52	0.42	11.01	0.59	-	-	26.11	0.41	3.76	0.63	22.67	0.38	2.85	0.6
Sup longitudinal fasc temporal R	28.75	0.36	-	-	-	-	6.44	0.38	-	-	11.15	0.33	-	-
**ICBM-DTI 81**	**% Vol**	**<ρ>**	**% Vol**	**<ρ>**	**% Vol**	**<ρ>**	**% Vol**	**<ρ>**	**% Vol**	**<ρ>**	**% Vol**	**<ρ>**	**% Vol**	**<ρ>**
Middle cerebellar peduncle	42.47	0.46	24.42	0.58	-	-	30.45	0.46	9.22	0.62	34.51	0.41	9.7	0.54
Pontine crossing tract (a part of MCP)	25.87	0.39	5.73	0.55	-	-	8.4	0.36	-	-	11.13	0.31	-	-
Genu of corpus callosum	24.51	0.35	-	-	-	-	-	-	-	-	20.6	0.34	-	-
Body of corpus callosum	27.62	0.36	2.1	0.53	-	-	17.42	0.37	-	-	11.87	0.32	1.48	0.49
Splenium of corpus callosum	29.72	0.42	13.34	0.57	-	-	12.76	0.39	-	-	26.07	0.42	12.06	0.55
Fornix (column and body of fornix)	70.26	0.53	60.55	0.61	-	-	56.6	0.4	-	-	66.31	0.41	-	-
Corticospinal tract R	27.61	0.39	9.25	0.56	-	-	9.54	0.43	-	-	29.3	0.34	-	-
Corticospinal tract L	39.64	0.42	17.66	0.57	-	-	18.1	0.41	6.93	0.52	39.85	0.4	9.93	0.54
Inferior cerebellar peduncle R	36.57	0.51	-	-	-	-	28.72	0.43	-	-	31.82	0.41	-	-
Inferior cerebellar peduncle L	31.61	0.51	-	-	-	-	28.31	0.45	-	-	30.68	0.39	-	-
Superior cerebellar peduncle R	9.17	0.31	-	-	-	-	2.22	0.33	-	-	-	-	-	-
Superior cerebellar peduncle L	21.57	0.33	-	-	-	-	9.98	0.3	-	-	13.41	0.35	-	-
Cerebral peduncle R	15.14	0.35	-	-	-	-	-	-	-	-	11.76	0.33	-	-
Cerebral peduncle L	25.81	0.35	-	-	-	-	5.18	0.36	-	-	16.55	0.36	3.07	0.56
Anterior limb of internal capsule L	17.86	0.36	-	-	-	-	5.2	0.33	-	-	21.64	0.35	-	-
Posterior limb of internal capsule R	5.59	0.34	-	-	-	-	4.1	0.36	-	-	-	-	-	-
Posterior limb of internal capsule L	4.13	0.36	-	-	-	-	3.41	0.35	-	-	1.25	0.31	-	-
Retrolenticular part of internal capsule L	24.75	0.41	8.59	0.57	-	-	9.23	0.38	1.62	0.52	15.07	0.38	1.3	0.53
Anterior corona radiata R	20.76	0.37	-	-	-	-	-	-	-	-	28.65	0.39	-	-
Anterior corona radiata L	31.67	0.4	3.49	0.57	-	-	4.71	0.39	-	-	35.55	0.41	8.73	0.58
Superior corona radiata R	10.91	0.36	-	-	-	-	3.04	0.38	-	-	2.96	0.36	-	-
Superior corona radiata L	11.97	0.41	-	-	-	-	5.35	0.42	-	-	6.15	0.35	-	-
Posterior corona radiata R	21.35	0.4	-	-	-	-	3.06	0.37	-	-	14.08	0.37	-	-
Posterior corona radiata L	25.01	0.42	7.35	0.54	-	-	14.14	0.38	-	-	17.07	0.4	-	-
Posterior thalamic radiation R	24.47	0.38	-	-	-	-	-	-	-	-	12.49	0.32	-	-
Posterior thalamic radiation L	47.23	0.41	16.16	0.54	-	-	32.91	0.4	-	-	23.63	0.34	-	-
Sagittal stratum R	23.52	0.37	-	-	-	-	10.95	0.35	-	-	11.71	0.31	-	-
Sagittal stratum L	53.2	0.47	34.02	0.6	5.6	0.77	46.35	0.46	24.47	0.57	34.2	0.35	6.59	0.56
External capsule R	41.85	0.39	11.12	0.53	-	-	19.98	0.37	-	-	20.8	0.33	-	-
External capsule L	56.33	0.47	31.04	0.59	-	-	39.07	0.42	6.23	0.53	28.98	0.38	5.55	0.57
Cingulum (cingulate gyrus) R	48.33	0.37	-	-	-	-	21.9	0.36	-	-	17.12	0.35	-	-
Cingulum (cingulate gyrus) L	51.73	0.43	20.61	0.57	-	-	30.39	0.41	-	-	31.3	0.38	-	-
Fornix (cres)/stria terminalis R	53.91	0.34	-	-	-	-	-	-	-	-	33.36	0.36	-	-
Fornix (cres)/stria terminalis L	63.56	0.45	41.33	0.54	-	-	34.04	0.37	-	-	55.02	0.39	12.71	0.59
Superior longitudinal fasciculus R	31.85	0.35	-	-	-	-	4.89	0.35	-	-	11.08	0.31	-	-
Superior longitudinal fasciculus L	54.26	0.42	14.46	0.58	-	-	41.56	0.42	9.9	0.58	21.62	0.36	-	-
Superior fronto-occipital fasciculus L	11.83	0.33	-	-	-	-	-	-	-	-	19.92	0.31	-	-
Uncinate fasciculus R	22.89	0.3	-	-	-	-	2.11	0.34	-	-	1.05	0.31	-	-
Uncinate fasciculus L	11.17	0.36	-	-	-	-	-	-	-	-	7.18	0.35	-	-
Tapetum R	51.01	0.36	-	-	-	-	-	-	-	-	49.66	0.34	-	-
Tapetum L	68.17	0.39	24.67	0.59	-	-	4.83	0.33	-	-	63.83	0.34	6.17	0.52

**Table 7:** Region of interest analysis was conducted for all free-water (fw) DTI metrics within the entire white matter. Significant group differences were identified in the f-index metric. All the data represent the mean ± standard deviation unless otherwise indicated. CN: cognitively normal. EMCI: Early Mild Cognitive Impairment. LMCI: Late Mild Cognitive Impairment. F: F-test. t: t-test. FA: fractional anisotropy. AX: axial diffusivity. RAD: radial diffusivity. *** *p* < 0.001.

**fw-FA**			
**Group**	**Mean**	**F-stat**	**Post hoc**
CN	0.412 ± 0.024	F = 0.101; *p* = 0.904	t = −0.178; *p* = 0.983
EMCI	0.412 ± 0.021		t = 0.380; *p* = 0.924
LMCI	0.411 ± 0.025		t = 0.448; *p* = 0.896
**fw-AX (×10^−3^)**			
**Group**	**Mean**	**F-stat**	**Post hoc**
CN	0.842 ± 0.053	F = 0.610; *p* = 0.543	t = 0.849; *p* = 0.673
EMCI	0.838 ± 0.046		t = −0.563; *p* = 0.840
LMCI	0.846 ± 0.046		t = −1.034; *p* = 0.556
**fw-RD (×10^−3^)**			
**Group**	**Mean**	**F-stat**	**Post hoc**
CN	0.426 ± 0.044	F = 0.392; *p* = 0.676	t = 0.170; *p* = 0.984
EMCI	0.425 ± 0.038		t = −0.830; *p* = 0.685
LMCI	0.431 ± 0.043		t = −0.839; *p* = 0.679
**f-index**			
**Group**	**Mean**	**F-stat**	**Post hoc**
CN	0.294 ± 0.041	F = 28.00; *p* < 0.001	t = −6.822; *p* < 0.001 ***
EMCI	0.321 ± 0.042		t = −4.128; *p* < 0.001 ***
LMCI	0.317 ± 0.040		t = 0.692; *p* = 0.768

**Table 8:** Volumetric analysis of white matter (WM), gray matter (GM), and cerebrospinal fluid (CSF) volumes across groups. The data are presented as the means (standard deviation). Shapiro–Wilk tests indicate non-normal distributions, and Kruskal–Wallis tests reveal significant differences across groups for all brain compartments. * Three CN individuals and one EMCI individual were excluded from this analysis due to the unavailability of structural images. ** *p* < 0.001.

Group	WM Volume (%)	GM Volume (%)	CSF Volume (%)
CN *	33.4 (2.7)	38.8 (3.1)	27.8 (3.5)
EMCI *	33.3 (2.7)	37.5 (2.8)	29.2 (1.8)
LMCI	31.8 (2.3)	36.6 (3.0)	31.7 (1.5)
**Shapiro–Wilk**	W = 0.973; *p* < 0.001	W = 0.971; *p* < 0.001	W = 0.967; *p* < 0.001
**Kruskal–Wallis**	Χ^2^ = 26.26; *p* < 0.001	Χ^2^ = 37.02; *p* < 0.001	Χ^2^ = 95.49; *p* < 0.001
Dunn test *			
CN-EMCI	Z = 0.54; *p* = 1.00	Z = 4.35; *p* < 0.001 **	Z = −3.92; *p* < 0.001 **
CN-LMCI	Z = 5.12; *p* < 0.001 **	Z = 4.90; *p* < 0.001 **	Z = −9.46; *p* < 0.001 **
EMCI-LMCI	Z = 4.17; *p* < 0.001 **	Z = 1.55; *p* = 0.363	Z = 5.85; *p* < 0.001 **

## Data Availability

Data are available on the ADNI database (https://adni.loni.usc.edu/).
